# Colour model analysis for microscopic image processing

**DOI:** 10.1186/1746-1596-3-S1-S18

**Published:** 2008-07-15

**Authors:** Gloria Bueno, Roberto González, Oscar Déniz, Jesús González, Marcial García-Rojo

**Affiliations:** 1 Escuela Técnica Superior de Ingenieros Industriales, Universidad de Castilla-La Mancha, Avda. Camilo José Cela, s/n, 13071 Ciudad Real, Spain; 2 Hospital General de Ciudad Real. Calle Tomelloso s/n. 13004 Ciudad Real, Spain

## Abstract

This article presents a comparative study between different colour models (RGB, HSI and CIEL*a*b*) applied to a very large microscopic image analysis. Such analysis of different colour models is needed in order to carry out a successful detection and therefore a classification of different regions of interest (ROIs) within the image. This, in turn, allows both distinguishing possible ROIs and retrieving their proper colour for further ROI analysis. This analysis is not commonly done in many biomedical applications that deal with colour images. Other important aspects is the computational cost of the different processing algorithms according to the colour model. This work takes these aspects into consideration to choose the best colour model tailored to the microscopic stain and tissue type under consideration and to obtain a successful processing of the histological image.

## Background

A challenge still facing scientists is the efficient analysis and management of biomedical data, including images. Advances in biomedical imaging diagnosis have been possible thanks to the development of new imaging technologies. Anatomical Pathology has also benefited from these new technologies, which have provided solutions for whole slide scanning by means of motorized microscopes and scanners [1], that is, whole slide imaging (WSI). However, the image processing performed with these slides is still limited both in data processed and processing methods.

Much research has been carried out on the development of algorithms for histological image analysis. Most of them are based on the segmentation of just one region of interest (ROI), which is usually the nucleus, and its classification for diagnosis purposes. To this end, statistical information techniques, region growing algorithms, active contour models and morphological methods have been used for ROI detection and processing [2-5].

The main problem with these methods is that they are not designed to process large amounts of data, which is the case when working with WSI in pathology. Besides that, many of these methods show limited results because they are mainly focused on a single structure or a type of tissue.

There is a need to develop more general and efficient image processing methods. To this end the colour model should be analysed, as well as the distance colour model applied to the processing algorithm in order to reduce the computational cost and obtain, in an efficient way, a set of heterogeneous, complex and specific image analysis. In this work different colour models and distances have been studied and applied under a general parallel image-processing model designed and implemented with MPP (Massively Parallel Processing).

## Methods

There are three main colour models:

RGB: channel Red, channel Green and channel Blue,

HSI: channel Hue, channel Saturation and channel Intensity,

L*a*b*: channel Luminance, channel a*, that is, range of channel between Red to Green and channel b* that is range of colours between Yellow to Blue.

All colour models have their advantages and drawbacks. It is necessary to identify which colour model is suitable to represent and reproduce the ROI under consideration for each tissue type and WSI modality. Analysing the distance colour formulae applied between two colours may do this, $d\left(\vec{x},\vec{y}\right)$, $\vec{x}=(x_{1,}x_{2},x_{3})$, $\vec{y}=(y_{1},y_{2},y_{3})$.

The distances considered within this study are: the Euclidean distance for the RGB model (Equation 1), the NBS colour distance formulae for HSI model (Equation 2) and the CIEDE2000 for the CIEL*a*b*, colour model (Equation 3).

(1) $$d\left(\vec{x},\vec{y}\right)=\sqrt{{\left(x_{1}-y_{1}\right)}^{2}+{\left(x_{2}-y_{2}\right)}^{2}+{\left(x_{3}-y_{3}\right)}^{2}}$$

(2) $$d\left(\vec{x},\vec{y}\right)=1.2*\sqrt{2x_{2}y_{2}\left(1-\cos\left(\frac{2\pi \Delta H}{100}\right)\right)+\Delta S^{2}+{\left(4\Delta I\right)}^{2}}$$

(3) $$\Delta E_{00}=\sqrt{{\left(\frac{\Delta L^{\prime }}{K_{L}S_{L}}\right)}^{2}+{\left(\frac{\Delta C^{\prime }}{K_{C}S_{C}}\right)}^{2}+{\left(\frac{\Delta H^{\prime }}{K_{H}S_{H}}\right)}^{2}+R_{T}\left(\frac{\Delta C^{\prime }}{K_{C}S_{C}}\right)\left(\frac{\Delta H^{\prime }}{K_{H}S_{H}}\right)}$$

Where *K*_*L*_, *K*_*c*_, *K*_*H *_are weight factors and the rest of components, *S*_*L*_, *S*_*C*_, *S*_*H*_, *C'*, *H'*, may be calculated by means of the, {L*, a*, b*} coordinates [6].

Moreover, another aspect to be considered is how to deal with the colour coordinates, that is as a vector or in a marginal way. These aspects have been analysed within this work. To this end the 3*2 distances to the most representative colour ROIs and statistically identified on the image were calculated on different WSI, which is to prostate biopsies and lung cytology stained with hematoxiline-eosine (HEO), inmunohistochemistry and papanicolau. The images were obtained by the ALIAS II automatic microscope and the processing was done using our own libraries, implemented by the research group, running under MPI on a grid composed by 17 nodes Intel XEON (3,2 GHz) INFINIBAND net (10 GB full-duplex) architecture. The results are shown as follows.

## Results

The results applied to microscopic images show that the Euclidean and NBS vector distance for the RGB and HSI model respectively distinguish between different ROIs but the vector CIEDE2000 distance for the CIEL*a*b* model reproduces in a better way, the original colour. However, the computational cost of the last one is higher than the other two colour models.

**Figure 1:**
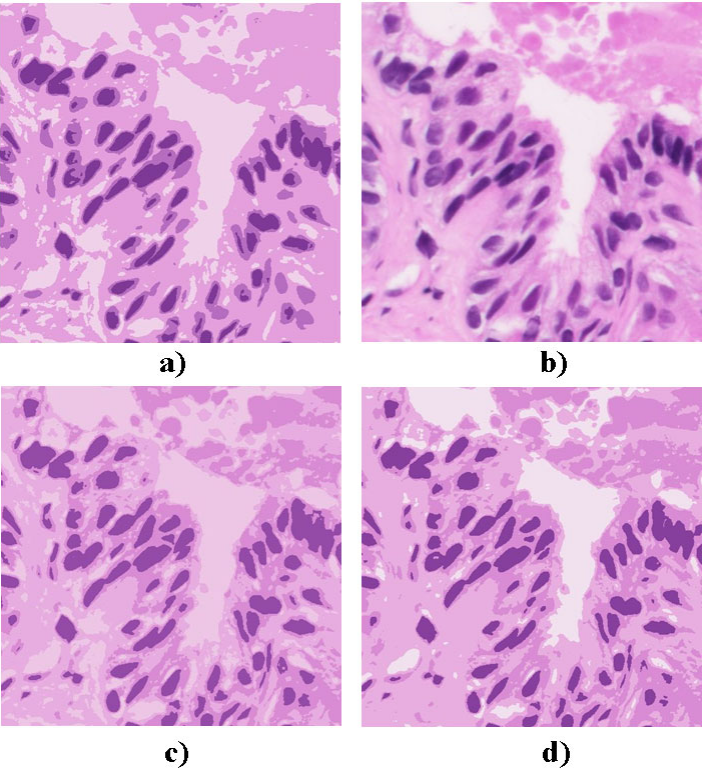
**Colour distances for ROI detection applied to biopsies**. a) Original Image, b) RGB (Euclidean), c) HSI (NBS), d) CIEL*a*b (CIEDE2000). Colour distances for ROI detection applied to biopsies.

**Figure 2:**
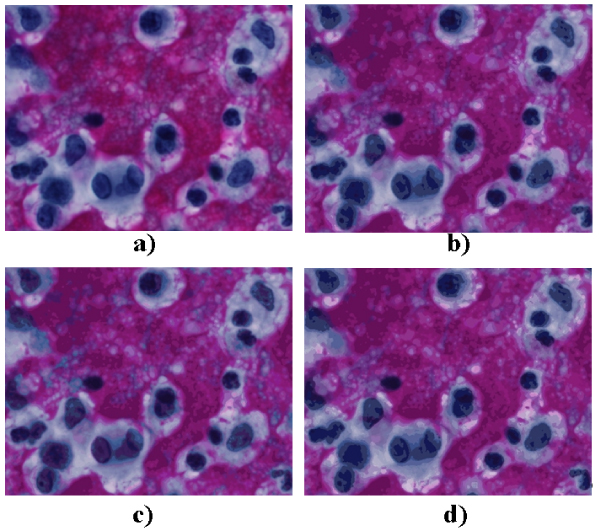
**Colour distances for ROI detection applied to cytology**. a) Original Image, b) RGB (Euclidean), c) HSI (NBS), d) CIEL*a*b (CIEDE2000). Colour distances for ROI detection applied to cytology.

Figure 1 shows the result for a biopsy stained with Hematoxiline-Eosine and Figure 2 for a cytology stained with Papanicolau.

**Figure 3:**
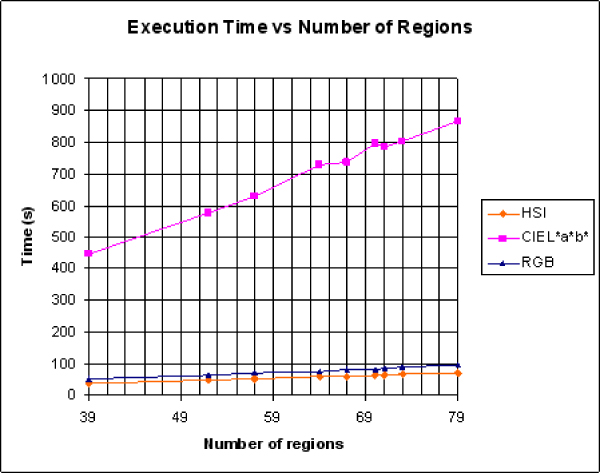
**Computational cost of the colour distances vs. number of ROIs**. Computational cost for the different colour models against the number of ROIs analysed.

The computational cost for the three colour distance vector models against different number of ROIs is shown in Figure 3.

**Figure 4:**
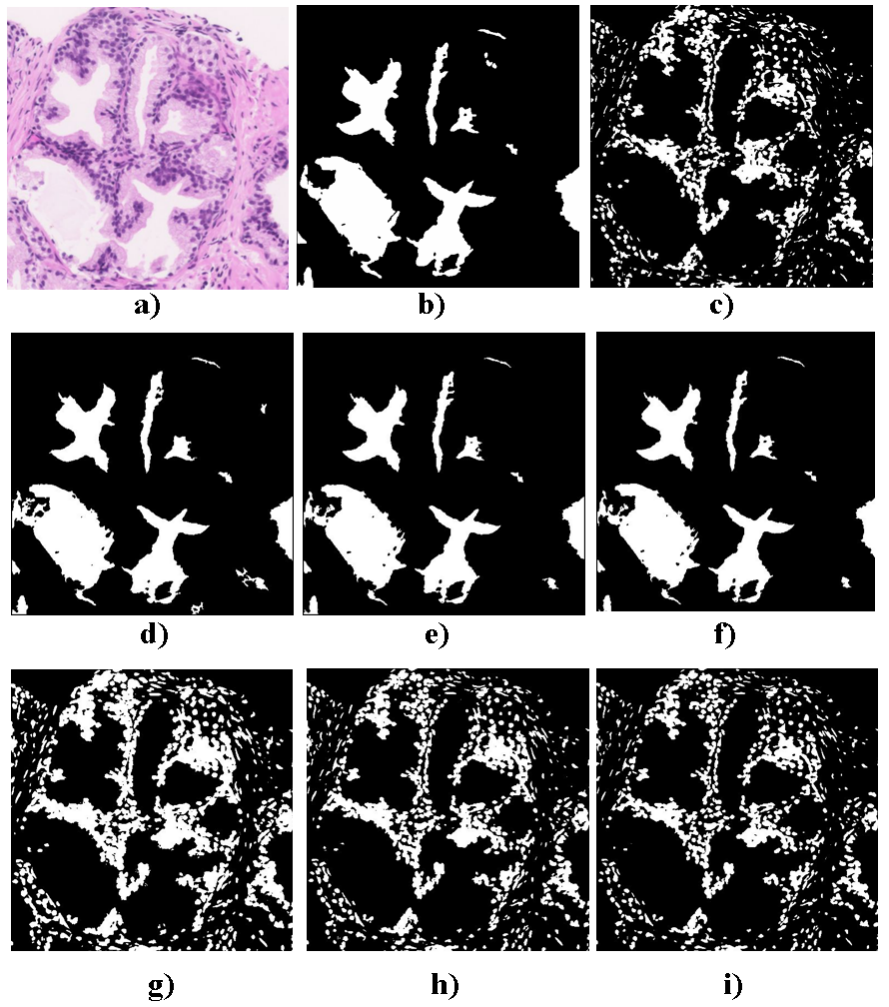
**Colour distance validation**. a) Original Image, b) True section of glandular light, c) True section of nucleus, d) RGB (Euclidean), e) HSI (NBS), f) CIEL*a*b (CIEDE2000), g) RGB (Euclidean), h) HSI (NBS), i) CIEL*a*b (CIEDE2000). Colour distances validation on different ROIs.

To quantify the goodness of the distance formulae a ROC analysis has been carried on. Figure 4 shows this analysis for two ROIs in a prostate biopsy at 10× stained with HEO. The true pixels belonging to the ROIS were indicated by experts at Hospital General Ciudad Real. Figure 4b) and 4c) show the true values for the two regions of interest, the glandular light and the nucleus, extracted from the original image (Figure 4a). Figure 4d) to i) show the different colour distance results for these two regions. Finally, Table 1 shows the ROC analysis for the Eucludian, NBS and CIEDE2000 colour distance for the RGB, HSI and CIEDEL*a*b* models. It is shown that the % of specificity is higher for the CIEDE2000 distance with lower value of FP.

**Table 1:** ROC analysis of the colour distance formulae for two ROIs

**GLANDULAR LIGHT**	**FP**	**FN**	**TP**	**Specificity**
**RGB **(Euclidean)	0.016563	0.066931	0.933069	0.983437
**HSI **(NBS)	0.006758	0.090020	0.909980	0.993242
CIEL*a*b* (CIEDE2000)	0.004757	0.100714	0.899286	0.995243

**NUCLEUS**	**FP**	**FN**	**TP**	**Specificity**

**RGB **(Euclidean)	0.154218	0.040697	0.959302	0.845782
**HSI **(NBS)	0.099283	0.109053	0.890947	0.900717
CIEL*a*b* (CIEDE2000)	0.057379	0.223000	0.777000	0.942621

## Conclusion

This article has presented a comparative study between RGB, HSI and CIEL*a*b* colour models applied histological images. This analysis, in turn, allows both distinguishing possible regions of interest and retrieving their proper colour for further region analysis.

The results applied to prostate biopsies stained with HEO and lung cytologies stained with papanicolau show that the vector CIEDE2000 distance for the CIEL*a*b* model reproduces in a better way the original colour.

Therefore, this comparison does allow us to choose the best colour model tailored to the microscopic stain and tissue type under consideration to obtain a successful processing. Moreover, a compromise between the computational cost and the results focus always to distinguish between different colour detection and colour retrieval for further ROI analysis should be kept. The colour model should be taken into consideration when defining standards for histological images.
